# Supplementary material for: p38-Mitogen Activated Kinases Mediate a Developmental Regulatory Response to Amino Acid Depletion and Associated Oxidative Stress in Mouse Blastocyst Embryos
Source: Front Cell Dev Biol. 2019 Nov 8;7:276. doi: 10.3389/fcell.2019.00276 (PMC6856562; doi:10.3389/fcell.2019.00276)
Supplement: FIGURE S1 — Projected confocal z-stack images of all blastocyst embryos stained with CellROX Green at E4.25 and imaged after fixation. [file Data_Sheet_1.pdf]

[illegible] Quantification of total and NANOG and/or GATA4 expressing cell numbers for all analysed embryos in KSOM +/- amino acids + DMSO or SB220025

KSOM

DMSO

#	TOTAL NUMBER OF CELLS							Ratio of PrE/Total ICM	Ratio of Inner/ Outer cells
			ICM						
	Embryo	Double Negative	NANOG +ve	GATA4 +ve	NANOG/ GATA4 double +ve	PrE	Total		
1	55	51	4	0	0	0	4	0.00	0.08
2	74	55	10	2	7	2	19	0.11	0.35
3	97	77	11	9	0	9	20	0.45	0.26
4	85	65	12	7	1	7	20	0.35	0.31
5	73	58	12	3	0	3	15	0.20	0.26
6	77	57	13	7	0	7	20	0.35	0.35
7	86	72	6	5	3	5	14	0.36	0.19
8	78	58	9	10	1	10	20	0.50	0.34
9	88	59	19	6	4	6	29	0.21	0.49
10	96	84	12	0	0	0	12	0.00	0.14
11	96	78	8	10	0	10	18	0.56	0.23
12	93	79	10	3	1	3	14	0.21	0.18
13	81	58	16	6	1	6	23	0.26	0.40
14	102	91	7	4	0	4	11	0.36	0.12
15	98	77	9	12	0	12	21	0.57	0.27
16	72	55	11	4	2	4	17	0.24	0.31
17	85	58	13	12	2	12	27	0.44	0.47
18	24	9	10	4	1	4	15	0.27	1.67
19	87	67	11	6	3	6	20	0.30	0.30
20	83	65	9	5	4	5	18	0.28	0.28
21	61	45	7	3	6	3	16	0.19	0.36
22	87	71	9	6	1	6	16	0.38	0.23
23	90	74	9	7	0	7	16	0.44	0.22
24	72	55	9	8	0	8	17	0.47	0.31
25	79	62	8	8	1	8	17	0.47	0.27
26	97	79	9	9	0	9	18	0.50	0.23
27	83	61	10	11	1	11	22	0.50	0.36
28	70	53	10	6	1	6	17	0.35	0.32
29	83	63	9	11	0	11	20	0.55	0.32
30	81	63	10	8	0	8	18	0.44	0.29
31	63	48	9	5	1	5	15	0.33	0.31
32	82	62	11	9	0	9	20	0.45	0.32
33	84	70	8	6	0	6	14	0.43	0.20
34	77	60	11	6	0	6	17	0.35	0.28
35	82	65	6	11	0	11	17	0.65	0.26
36	106	86	9	11	0	11	20	0.55	0.23
37	92	68	9	14	1	14	24	0.58	0.35
38	101	79	12	10	0	10	22	0.45	0.28
39	118	91	15	12	0	12	27	0.44	0.30
40	75	63	4	5	3	5	12	0.42	0.19
41	65	51	12	2	0	2	14	0.14	0.27
42	70	60	5	3	2	3	10	0.30	0.17
43	65	52	6	2	5	2	13	0.15	0.25
44	77	63	8	5	1	5	14	0.36	0.22
45	92	79	8	4	1	4	13	0.31	0.16
46	48	39	4	0	5	0	9	0.00	0.23
47	79	61	9	8	1	8	18	0.44	0.30
48	81	61	12	8	0	8	20	0.40	0.33
49	67	55	7	5	0	5	12	0.42	0.22
50	83	66	11	6	0	6	17	0.35	0.26
51	60	54	4	2	0	2	6	0.33	0.11
52	44	31	12	0	1	0	13	0.00	0.42
53	108	79	16	11	2	11	29	0.38	0.37
54	75	53	10	9	3	9	22	0.41	0.42
55	70	59	10	0	1	0	11	0.00	0.19
56	75	60	5	9	1	9	15	0.60	0.25
57	73	50	15	8	0	8	23	0.35	0.46
58	93	63	16	10	4	10	30	0.33	0.48
59	79	61	12	4	2	4	18	0.22	0.30
60	70	52	8	10	0	10	18	0.56	0.35
61	97	78	11	5	3	5	19	0.26	0.24
62	89	73	9	7	0	7	16	0.44	0.22
63	82	67	11	2	2	2	15	0.13	0.22
64	92	80	10	1	1	1	12	0.08	0.15
65	82	55	15	10	2	10	27	0.37	0.49
66	87	60	24	3	0	3	27	0.11	0.45
67	88	65	9	11	3	11	23	0.48	0.35
68									
69									
70									
71									
72									
73									
74									
75									
76									
77									
78									
79									
80									
**TOTAL**	**5404**	**4218**	**675**	**426**	**85**	**426**	**1186**	22.89	20.48
**AVERAGE**	**80.7**	**63.0**	**10.1**	**6.4**	**1.3**	**6.4**	**17.7**	0.34	0.31
**SEM**	**1.9**	**1.6**	**0.4**	**0.4**	**0.2**	**0.4**	**0.6**	0.02	0.02

p38 Inhibited

#	TOTAL NUMBER OF CELLS							Ratio of PrE/Total ICM	Ratio of Inner/ Outer cells
			ICM						
	Embryo	Double Negative	NANOG +ve	GATA4 +ve	NANOG/ GATA4 double +ve	PrE	Total		
1	15	15	0	0	0	0	0	0.00	0.00
2	28	27	1	0	0	0	1	0.00	0.04
3	45	40	4	1	0	1	5	0.20	0.13
4	65	53	8	3	1	3	12	0.25	0.23
5	41	37	4	0	0	0	4	0.00	0.11
6	38	35	3	0	0	0	3	0.00	0.09
7	35	25	9	1	0	1	10	0.10	0.40
8	49	41	7	1	0	1	8	0.13	0.20
9	29	26	3	0	0	0	3	0.00	0.12
10	27	22	5	0	0	0	5	0.00	0.23
11	34	29	5	0	0	0	5	0.00	0.17
12	32	30	2	0	0	0	2	0.00	0.07
13	69	49	16	4	0	4	20	0.20	0.41
14	61	51	6	4	0	4	10	0.40	0.20
15	78	67	5	6	0	6	11	0.55	0.16
16	41	40	1	0	0	0	1	0.00	0.03
17	37	31	4	0	2	0	6	0.00	0.19
18	54	45	5	3	1	3	9	0.33	0.20
19	41	37	3	0	1	0	4	0.00	0.11
20	37	34	2	0	1	0	3	0.00	0.09
21	31	19	11	1	0	1	12	0.08	0.63
22	31	29	2	0	0	0	2	0.00	0.07
23	27	17	10	0	0	0	10	0.00	0.59
24	22	18	4	0	0	0	4	0.00	0.22
25	36	30	6	0	0	0	6	0.00	0.20
26	59	50	8	1	0	1	9	0.11	0.18
27	53	46	5	1	1	1	7	0.14	0.15
28	49	36	9	4	0	4	13	0.31	0.36
29	57	51	4	2	0	2	6	0.33	0.12
30	70	56	8	6	0	6	14	0.43	0.25
31	62	50	12	0	0	0	12	0.00	0.24
32	43	36	5	2	0	2	7	0.29	0.19
33	33	27	5	1	0	1	6	0.17	0.22
34	22	19	3	0	0	0	3	0.00	0.16
35	39	33	5	1	0	1	6	0.17	0.18
36	67	60	3	2	2	2	7	0.29	0.12
37	60	40	15	5	0	5	20	0.25	0.50
38	45	41	4	0	0	0	4	0.00	0.10
39	30	26	3	1	0	1	4	0.25	0.15
40	65	56	6	1	2	1	9	0.11	0.16
41	68	60	8	0	0	0	8	0.00	0.13
42	45	34	11	0	0	0	11	0.00	0.32
43	58	54	4	0	0	0	4	0.00	0.07
44	57	49	5	3	0	3	8	0.38	0.16
45	70	65	3	0	2	0	5	0.00	0.08
46	37	35	2	0	0	0	2	0.00	0.06
47	75	67	6	2	0	2	8	0.25	0.12
48	29	18	11	0	0	0	11	0.00	0.61
49	50	43	6	1	0	1	7	0.14	0.16
50	25	16	9	0	0	0	9	0.00	0.56
51	32	29	3	0	0	0	3	0.00	0.10
52	25	22	3	0	0	0	3	0.00	0.14
53	29	25	4	0	0	0	4	0.00	0.16
54	54	48	6	0	0	0	6	0.00	0.13
55	37	30	7	0	0	0	7	0.00	0.23
56	28	27	1	0	0	0	1	0.00	0.04
57	12	12	0	0	0	0	0	0.00	0.00
58	31	27	2	2	0	2	4	0.50	0.15
59	50	38	11	0	1	0	12	0.00	0.32
60	66	58	8	0	0	0	8	0.00	0.14
61	44	38	6	0	0	0	6	0.00	0.16
62	50	46	4	0	0	0	4	0.00	0.09
63	39	35	4	0	0	0	4	0.00	0.11
64	76	72	4	0	0	0	4	0.00	0.06
65	34	31	3	0	0	0	3	0.00	0.10
66	55	52	3	0	0	0	3	0.00	0.06
67	39	34	5	0	0	0	5	0.00	0.15
68	31	24	7	0	0	0	7	0.00	0.29
69	38	21	17	0	0	0	17	0.00	0.81
70	38	29	9	0	0	0	9	0.00	0.31
71	90	84	5	1	0	1	6	0.17	0.07
72	45	37	8	0	0	0	8	0.00	0.22
73	27	16	11	0	0	0	11	0.00	0.69
74	40	35	5	0	0	0	5	0.00	0.14
75	37	30	5	0	2	0	7	0.00	0.23
76	52	43	9	0	0	0	9	0.00	0.21
77	45	34	11	0	0	0	11	0.00	0.32
78									
79									
80									
**TOTAL**	**3415**	**2892**	**447**	**60**	**16**	**60**	**523**	6.51	15.63
**AVERAGE**	**44.4**	**37.6**	**5.8**	**0.8**	**0.2**	**0.8**	**6.8**	0.08	0.20
**SEM**	**1.8**	**1.7**	**0.4**	**0.2**	**0.1**	**0.2**	**0.5**	0.02	0.02

Supplementary table S1 (contd.)

KSOM with amino acids

DMSO

#	TOTAL NUMBER OF CELLS							Ratio of PrE/Total ICM	Ratio of Inner/ Outer cells
			ICM						
	Embryo	Double Negative	NANOG +ve	GATA4 +ve	NANOG/ GATA4 double +ve	PrE	Total		
1	52	34	17	0	1	0	18	0.00	0.53
2	84	67	9	8	0	8	17	0.47	0.25
3	68	51	15	2	0	2	17	0.12	0.33
4	83	69	7	7	0	7	14	0.50	0.20
5	66	57	8	1	0	1	9	0.11	0.16
6	87	73	9	5	0	5	14	0.36	0.19
7	59	50	8	1	0	1	9	0.11	0.18
8	74	62	11	1	0	1	12	0.08	0.19
9	81	59	16	4	2	4	22	0.18	0.37
10	66	48	10	7	1	7	18	0.39	0.38
11	75	61	7	7	0	7	14	0.50	0.23
12	61	48	7	6	0	6	13	0.46	0.27
13	78	64	10	3	1	3	14	0.21	0.22
14	74	60	6	8	0	8	14	0.57	0.23
15	71	48	11	12	0	12	23	0.52	0.48
16	78	62	7	8	1	8	16	0.50	0.26
17	79	66	6	4	3	4	13	0.31	0.20
18	84	61	10	12	1	12	23	0.52	0.38
19	44	35	4	3	2	3	9	0.33	0.26
20	91	70	14	7	0	7	21	0.33	0.30
21	76	67	5	3	1	3	9	0.33	0.13
22	78	63	10	5	0	5	15	0.33	0.24
23	93	75	10	7	1	7	18	0.39	0.24
24	96	66	16	14	0	14	30	0.47	0.45
25	76	48	11	17	0	17	28	0.61	0.58
26	80	59	8	13	0	13	21	0.62	0.36
27	90	80	5	5	0	5	10	0.50	0.13
28	72	57	10	5	0	5	15	0.33	0.26
29	83	64	12	7	0	7	19	0.37	0.30
30	95	69	13	12	1	12	26	0.46	0.38
31	82	62	10	9	1	9	20	0.45	0.32
32	69	58	8	2	1	2	11	0.18	0.19
33	88	71	13	4	0	4	17	0.24	0.24
34	77	66	3	8	0	8	11	0.73	0.17
35	84	60	9	15	0	15	24	0.63	0.40
36	86	62	14	9	1	9	24	0.38	0.39
37	89	70	13	6	0	6	19	0.32	0.27
38	72	58	11	1	2	1	14	0.07	0.24
39	58	48	6	4	0	4	10	0.40	0.21
40	73	52	6	15	0	15	21	0.71	0.40
41	85	63	15	5	2	5	22	0.23	0.35
42	90	67	7	15	1	15	23	0.65	0.34
43	85	67	9	7	2	7	18	0.39	0.27
44	117	98	11	7	1	7	19	0.37	0.19
45	84	74	6	4	0	4	10	0.40	0.14
46	88	69	10	8	1	8	19	0.42	0.28
47	107	89	11	7	0	7	18	0.39	0.20
48	98	68	12	16	2	16	30	0.53	0.44
49	94	81	11	1	1	1	13	0.08	0.16
50	90	67	16	7	0	7	23	0.30	0.34
51	80	60	10	8	2	8	20	0.40	0.33
52	79	57	8	12	2	12	22	0.55	0.39
53	93	71	19	3	0	3	22	0.14	0.31
54	66	45	18	3	0	3	21	0.14	0.47
55	101	74	14	11	2	11	27	0.41	0.36
56	72	55	12	5	0	5	17	0.29	0.31
57	83	57	15	9	2	9	26	0.35	0.46
58	71	40	21	5	5	5	31	0.16	0.78
59	92	73	8	7	4	7	19	0.37	0.26
60	78	58	11	3	6	3	20	0.15	0.34
61	80	60	13	7	0	7	20	0.35	0.33
62	77	56	10	11	0	11	21	0.52	0.38
63	60	40	20	0	0	0	20	0.00	0.50
64	78	69	6	3	0	3	9	0.33	0.13
65	95	75	8	9	3	9	20	0.45	0.27
66	106	88	13	4	1	4	18	0.22	0.20
67	80	62	8	8	2	8	18	0.44	0.29
68	111	82	12	17	0	17	29	0.59	0.35
69	110	88	10	12	0	12	22	0.55	0.25
70	76	59	10	7	0	7	17	0.41	0.29
71	96	76	10	10	0	10	20	0.50	0.26
72									
73									
74									
75									
76									
77									
78									
79									
80									
**TOTAL**	**5794**	**4488**	**749**	**498**	**59**	**498**	**1306**	26.17	21.48
**AVERAGE**	**81.6**	**63.2**	**10.5**	**7.0**	**0.8**	**7.0**	**18.4**	0.37	0.30
**SEM**	**1.6**	**1.5**	**0.4**	**0.5**	**0.1**	**0.5**	**0.7**	0.02	0.01

p38 Inhibited

#	TOTAL NUMBER OF CELLS							Ratio of PrE/Total ICM	Ratio of Inner/ Outer cells
			ICM						
	Embryo	Double Negative	NANOG +ve	GATA4 +ve	NANOG/ GATA4 double +ve	PrE	Total		
1	79	71	7	0	1	0	8	0.00	0.11
2	66	55	8	3	0	3	11	0.27	0.20
3	63	49	11	3	0	3	14	0.21	0.29
4	59	53	5	0	1	0	6	0.00	0.11
5	52	45	6	1	0	1	7	0.14	0.16
6	54	47	5	0	2	0	7	0.00	0.15
7	56	49	6	1	0	1	7	0.14	0.14
8	46	38	8	0	0	0	8	0.00	0.21
9	47	42	5	0	0	0	5	0.00	0.12
10	53	43	6	3	1	3	10	0.30	0.23
11	36	27	6	1	2	1	9	0.11	0.33
12	70	56	10	3	1	3	14	0.21	0.25
13	55	46	7	2	0	2	9	0.22	0.20
14	46	38	5	0	3	0	8	0.00	0.21
15	56	49	5	1	1	1	7	0.14	0.14
16	47	37	9	1	0	1	10	0.10	0.27
17	62	52	7	3	0	3	10	0.30	0.19
18	57	42	14	1	0	1	15	0.07	0.36
19	55	44	9	1	1	1	11	0.09	0.25
20	51	42	9	0	0	0	9	0.00	0.21
21	53	42	8	2	1	2	11	0.18	0.26
22	78	60	9	8	1	8	18	0.44	0.30
23	61	56	5	0	0	0	5	0.00	0.09
24	45	40	5	0	0	0	5	0.00	0.13
25	51	37	14	0	0	0	14	0.00	0.38
26	45	34	10	1	0	1	11	0.09	0.32
27	53	46	5	1	1	1	7	0.14	0.15
28	62	51	8	3	0	3	11	0.27	0.22
29	55	40	9	5	1	5	15	0.33	0.38
30	58	50	6	2	0	2	8	0.25	0.16
31	64	54	7	0	3	0	10	0.00	0.19
32	78	65	11	2	0	2	13	0.15	0.20
33	65	48	9	8	0	8	17	0.47	0.35
34	35	29	6	0	0	0	6	0.00	0.21
35	73	53	15	5	0	5	20	0.25	0.38
36	71	48	23	0	0	0	23	0.00	0.48
37	77	57	10	10	0	10	20	0.50	0.35
38	61	50	10	1	0	1	11	0.09	0.22
39	75	65	7	3	0	3	10	0.30	0.15
40	62	43	16	3	0	3	19	0.16	0.44
41	61	47	12	2	0	2	14	0.14	0.30
42	79	66	9	4	0	4	13	0.31	0.20
43	23	18	5	0	0	0	5	0.00	0.28
44	68	54	9	4	1	4	14	0.29	0.26
45	68	59	5	4	0	4	9	0.44	0.15
46	57	43	9	5	0	5	14	0.36	0.33
47	63	50	13	0	0	0	13	0.00	0.26
48	64	59	5	0	0	0	5	0.00	0.08
49	66	55	10	1	0	1	11	0.09	0.20
50	52	44	4	3	1	3	8	0.38	0.18
51	68	61	7	0	0	0	7	0.00	0.11
52	66	50	15	1	0	1	16	0.06	0.32
53	60	49	7	2	2	2	11	0.18	0.22
54	49	37	12	0	0	0	12	0.00	0.32
55	57	50	7	0	0	0	7	0.00	0.14
56	85	60	16	8	1	8	25	0.32	0.42
57	63	54	8	0	1	0	9	0.00	0.17
58	71	60	11	0	0	0	11	0.00	0.18
59	53	34	18	0	1	0	19	0.00	0.56
60	51	48	3	0	0	0	3	0.00	0.06
61	49	39	9	1	0	1	10	0.10	0.26
62	35	27	7	1	0	1	8	0.13	0.30
63	45	28	17	0	0	0	17	0.00	0.61
64	71	59	8	1	3	1	12	0.08	0.20
65	55	49	5	1	0	1	6	0.17	0.12
66	59	29	30	0	0	0	30	0.00	1.03
67	43	24	19	0	0	0	19	0.00	0.79
68	52	44	5	2	1	2	8	0.25	0.18
69	55	34	20	0	1	0	21	0.00	0.62
70	66	56	10	0	0	0	10	0.00	0.18
71	65	50	11	4	0	4	15	0.27	0.30
72	62	49	11	2	0	2	13	0.15	0.27
73	48	36	12	0	0	0	12	0.00	0.33
74	85	69	6	10	0	10	16	0.63	0.23
75	73	56	14	1	2	1	17	0.06	0.30
76	76	54	11	11	0	11	22	0.50	0.41
77									
78									
79									
80									
**TOTAL**	**4495**	**3594**	**721**	**146**	**34**	**146**	**901**	10.86	20.37
**AVERAGE**	**59.1**	**47.3**	**9.5**	**1.9**	**0.4**	**1.9**	**11.9**	0.14	0.27
**SEM**	**1.4**	**1.2**	**0.5**	**0.3**	**0.1**	**0.3**	**0.6**	0.02	0.02

Supplementary table S2: Quantification of total and NANOG and/or GATA4 expressing cell numbers for all analysed embryos in KSOM +/- amino acids + DMSO or SB220025 + 1 or 10mM NAC

KSOM + 1mM NAC

DMSO

#	TOTAL NUMBER OF CELLS							Ratio of PrE/Total ICM	Ratio of Inner/ Outer cells
	Embryo	Double Negative	ICM						
			NANOG +ve	GATA4 +ve	NANOG/ GATA4 double +ve	PrE	Total		
1	82	63	8	11	0	11	19	0.58	0.30
2	65	63	2	0	0	0	2	0.00	0.03
3	38	28	9	0	1	0	10	0.00	0.36
4	70	49	11	10	0	10	21	0.48	0.43
5	70	60	6	4	0	4	10	0.40	0.17
6	71	53	10	8	0	8	18	0.44	0.34
7	89	73	10	6	0	6	16	0.38	0.22
8	83	70	5	6	2	6	13	0.46	0.19
9	95	80	7	8	0	8	15	0.53	0.19
10	96	82	9	5	0	5	14	0.36	0.17
11	88	73	9	6	0	6	15	0.40	0.21
12	74	68	3	3	0	3	6	0.50	0.09
13	85	60	12	13	0	13	25	0.52	0.42
14	98	69	16	13	0	13	29	0.45	0.42
15	111	79	21	11	0	11	32	0.34	0.41
16	80	65	12	3	0	3	15	0.20	0.23
17	77	57	14	5	1	5	20	0.25	0.35
18	97	75	13	8	1	8	22	0.36	0.29
19	73	51	17	2	3	2	22	0.09	0.43
20	70	43	14	10	3	10	27	0.37	0.63
21	91	68	13	10	0	10	23	0.43	0.34
22	62	50	7	2	3	2	12	0.17	0.24
23	75	47	18	9	1	9	28	0.32	0.60
24	91	73	10	7	1	7	18	0.39	0.25
25	64	52	6	6	0	6	12	0.50	0.23
26	84	71	8	4	1	4	13	0.31	0.18
27	77	58	11	8	0	8	19	0.42	0.33
28	84	70	9	5	0	5	14	0.36	0.20
29									
30									
31									
32									
33									
34									
35									
36									
37									
38									
39									
40									
TOTAL	2240	1750	290	183	17	183	490	10.01	8.22
AVERAGE	80.0	62.5	10.4	6.5	0.6	6.5	17.5	0.36	0.29
SEM	2.7	2.4	0.8	0.7	0.2	0.7	1.3	0.03	0.03

p38 Inhibited

#	TOTAL NUMBER OF CELLS							Ratio of PrE/Total ICM	Ratio of Inner/ Outer cells
	Embryo	Double Negative	ICM						
			NANOG +ve	GATA4 +ve	NANOG/ GATA4 double +ve	PrE	Total		
1	43	41	2	0	0	0	2	0.00	0.05
2	42	36	4	2	0	2	6	0.33	0.17
3	46	43	3	0	0	0	3	0.00	0.07
4	63	56	5	2	0	2	7	0.29	0.13
5	58	48	10	0	0	0	10	0.00	0.21
6	27	16	11	0	0	0	11	0.00	0.69
7	38	36	2	0	0	0	2	0.00	0.06
8	56	48	8	0	0	0	8	0.00	0.17
9	34	25	6	3	0	3	9	0.33	0.36
10	51	37	9	5	0	5	14	0.36	0.38
11	29	11	18	0	0	0	18	0.00	1.64
12	48	43	3	2	0	2	5	0.40	0.12
13	34	30	4	0	0	0	4	0.00	0.13
14	35	29	6	0	0	0	6	0.00	0.21
15	52	46	2	3	1	3	6	0.50	0.13
16	58	47	9	2	0	2	11	0.18	0.23
17	41	34	5	2	0	2	7	0.29	0.21
18	67	53	8	6	0	6	14	0.43	0.26
19	67	62	5	0	0	0	5	0.00	0.08
20	70	54	14	2	0	2	16	0.13	0.30
21	77	63	9	5	0	5	14	0.36	0.22
22	43	29	11	1	2	1	14	0.07	0.48
23	58	50	6	0	2	0	8	0.00	0.16
24	92	78	9	4	1	4	14	0.29	0.18
25	71	61	5	5	0	5	10	0.50	0.16
26	55	46	7	2	0	2	9	0.22	0.20
27	47	40	6	0	1	0	7	0.00	0.18
28	78	67	11	0	0	0	11	0.00	0.16
29	83	81	2	0	0	0	2	0.00	0.02
30	81	70	7	4	0	4	11	0.36	0.16
31	47	46	1	0	0	0	1	0.00	0.02
32	45	38	7	0	0	0	7	0.00	0.18
33	75	67	4	4	0	4	8	0.50	0.12
34	65	61	2	2	0	2	4	0.50	0.07
35	60	51	9	0	0	0	9	0.00	0.18
36	70	63	4	3	0	3	7	0.43	0.11
37	60	51	9	0	0	0	9	0.00	0.18
38	59	59	0	0	0	0	0	0.00	0.00
39									
40									
TOTAL	2125	1816	243	59	7	59	309	6.46	8.35
AVERAGE	55.9	47.8	6.4	1.6	0.2	1.6	8.1	0.17	0.22
SEM	2.6	2.6	0.6	0.3	0.1	0.3	0.7	0.03	0.04

KSOM + 10mM NAC

DMSO

#	TOTAL NUMBER OF CELLS							Ratio of PrE/Total ICM	Ratio of Inner/ Outer cells
	Embryo	Double Negative	ICM						
			NANOG +ve	GATA4 +ve	NANOG/ GATA4 double +ve	PrE	Total		
1	57	39	10	8	0	8	18	0.44	0.46
2	75	53	16	6	0	6	22	0.27	0.42
3	74	58	9	7	0	7	16	0.44	0.28
4	77	59	15	3	0	3	18	0.17	0.31
5	43	38	5	0	0	0	5	0.00	0.13
6	57	48	3	5	1	5	9	0.56	0.19
7	51	40	11	0	0	0	11	0.00	0.28
8	55	41	10	4	0	4	14	0.29	0.34
9	103	83	10	8	2	8	20	0.40	0.24
10	81	60	10	11	0	11	21	0.52	0.35
11	82	61	9	11	1	11	21	0.52	0.34
12	95	78	7	10	0	10	17	0.59	0.22
13	83	72	8	3	0	3	11	0.27	0.15
14	93	75	9	9	0	9	18	0.50	0.24
15	83	66	9	8	0	8	17	0.47	0.26
16	102	86	7	9	0	9	16	0.56	0.19
17	72	48	12	12	0	12	24	0.50	0.50
18									
19									
20									
TOTAL	1283	1005	160	114	4	114	278	6.50	4.88
AVERAGE	75.5	59.1	9.4	6.7	0.2	6.7	16.4	0.38	0.29
SEM	4.3	3.8	0.8	0.9	0.1	0.9	1.2	0.05	0.03

p38 Inhibited

#	TOTAL NUMBER OF CELLS							Ratio of PrE/Total ICM	Ratio of Inner/ Outer cells
	Embryo	Double Negative	ICM						
			NANOG +ve	GATA4 +ve	NANOG/ GATA4 double +ve	PrE	Total		
1	30	27	3	0	0	0	3	0.00	0.11
2	61	56	2	3	0	3	5	0.60	0.09
3	45	35	6	4	0	4	10	0.40	0.29
4	52	37	9	2	4	2	15	0.13	0.41
5	40	32	4	4	0	4	8	0.50	0.25
6	50	44	6	0	0	0	6	0.00	0.14
7	49	45	4	0	0	0	4	0.00	0.09
8	52	39	6	6	1	6	13	0.46	0.33
9	68	55	9	4	0	4	13	0.31	0.24
10	90	74	8	8	0	8	16	0.50	0.22
11	69	61	8	0	0	0	8	0.00	0.13
12	78	59	13	6	0	6	19	0.32	0.32
13	62	52	10	0	0	0	10	0.00	0.19
14	47	39	6	2	0	2	8	0.25	0.21
15	81	67	8	6	0	6	14	0.43	0.21
16	72	64	7	1	0	1	8	0.13	0.13
17	52	45	5	2	0	2	7	0.29	0.16
18	36	25	8	3	0	3	11	0.27	0.44
19									
20									
TOTAL	1034	856	122	51	5	51	178	4.58	3.93
AVERAGE	57.4	47.6	6.8	2.8	0.3	2.8	9.9	0.25	0.22
SEM	3.8	3.3	0.6	0.6	0.2	0.6	1.0	0.05	0.02

Supplementary table S2 (contd.)

KSOM+AA + 1mM NAC

DMSO

#	TOTAL NUMBER OF CELLS							Ratio of PrE/Total ICM	Ratio of Inner/ Outer cells
	Embryo	Double Negative	ICM						
			NANOG +ve	GATA4 +ve	NANOG/ GATA4 double +ve	PrE	Total		
1	97	75	12	9	1	9	22	0.41	0.29
2	87	77	7	1	2	1	10	0.10	0.13
3	49	33	10	3	3	3	16	0.19	0.48
4	45	30	11	1	3	1	15	0.07	0.50
5	68	56	9	2	1	2	12	0.17	0.21
6	78	50	17	11	0	11	28	0.39	0.56
7	78	62	10	5	1	5	16	0.31	0.26
8	56	53	3	0	0	0	3	0.00	0.06
9	68	45	16	5	2	5	23	0.22	0.51
10	91	73	6	12	0	12	18	0.67	0.25
11	78	57	13	8	0	8	21	0.38	0.37
12	89	68	5	12	4	12	21	0.57	0.31
13	93	77	9	7	0	7	16	0.44	0.21
14	88	63	6	18	1	18	25	0.72	0.40
15	73	47	13	9	4	9	26	0.35	0.55
16	67	46	19	2	0	2	21	0.10	0.46
17	79	67	9	3	0	3	12	0.25	0.18
18	100	76	13	11	0	11	24	0.46	0.32
19	93	75	9	8	1	8	18	0.44	0.24
20	90	66	17	7	0	7	24	0.29	0.36
21									
22									
23									
24									
25									
26									
27									
28									
29									
30									
31									
32									
33									
34									
35									
36									
37									
38									
39									
40									
TOTAL	1567	1196	214	134	23	134	371	6.52	6.64
AVERAGE	78.4	59.8	10.7	6.7	1.2	6.7	18.6	0.33	0.33
SEM	3.5	3.2	1.0	1.1	0.3	1.1	1.4	0.04	0.03

p38 Inhibited

#	TOTAL NUMBER OF CELLS							Ratio of PrE/Total ICM	Ratio of Inner/ Outer cells
	Embryo	Double Negative	ICM						
			NANOG +ve	GATA4 +ve	NANOG/ GATA4 double +ve	PrE	Total		
1	79	68	5	6	0	6	11	0.55	0.16
2	57	43	12	2	0	2	14	0.14	0.33
3	63	42	15	5	1	5	21	0.24	0.50
4	66	57	9	0	0	0	9	0.00	0.16
5	60	45	12	3	0	3	15	0.20	0.33
6	81	73	2	6	0	6	8	0.75	0.11
7	95	79	9	6	1	6	16	0.38	0.20
8	59	46	13	0	0	0	13	0.00	0.28
9	13	8	5	0	0	0	5	0.00	0.63
10	76	68	3	5	0	5	8	0.63	0.12
11	28	11	17	0	0	0	17	0.00	1.55
12	96	84	8	4	0	4	12	0.33	0.14
13	76	61	9	4	2	4	15	0.27	0.25
14	71	55	16	0	0	0	16	0.00	0.29
15	45	37	8	0	0	0	8	0.00	0.22
16	58	44	14	0	0	0	14	0.00	0.32
17	85	72	13	0	0	0	13	0.00	0.18
18	60	47	11	2	0	2	13	0.15	0.28
19	80	71	6	3	0	3	9	0.33	0.13
20	63	55	7	1	0	1	8	0.13	0.15
21	62	49	5	8	0	8	13	0.62	0.27
22	22	19	3	0	0	0	3	0.00	0.16
23									
24									
25									
26									
27									
28									
29									
30									
31									
32									
33									
34									
35									
36									
37									
38									
39									
40									
TOTAL	1395	1134	202	55	4	55	261	4.70	6.73
AVERAGE	63.4	51.5	9.2	2.5	0.2	2.5	11.9	0.21	0.31
SEM	4.6	4.4	0.9	0.6	0.1	0.6	0.9	0.05	0.06

KSOM+AA + 10mM NAC

DMSO

#	TOTAL NUMBER OF CELLS							Ratio of PrE/Total ICM	Ratio of Inner/ Outer cells
	Embryo	Double Negative	ICM						
			NANOG +ve	GATA4 +ve	NANOG/ GATA4 double +ve	PrE	Total		
1	84	61	17	6	0	6	23	0.26	0.38
2	64	54	9	1	0	1	10	0.10	0.19
3	66	41	21	3	1	3	25	0.12	0.61
4	66	40	21	1	4	1	26	0.04	0.65
5	95	67	16	12	0	12	28	0.43	0.42
6	58	38	16	3	1	3	20	0.15	0.53
7	82	65	7	10	0	10	17	0.59	0.26
8	92	76	6	10	0	10	16	0.63	0.21
9	45	35	5	4	1	4	10	0.40	0.29
10	70	55	7	7	1	7	15	0.47	0.27
11	68	57	7	4	0	4	11	0.36	0.19
12	57	45	7	3	2	3	12	0.25	0.27
13	91	75	9	7	0	7	16	0.44	0.21
14	93	78	8	6	1	6	15	0.40	0.19
15	84	64	8	11	1	11	20	0.55	0.31
16	85	65	10	8	2	8	20	0.40	0.31
17	69	59	10	0	0	0	10	0.00	0.17
18	71	56	7	6	2	6	15	0.40	0.27
19	94	72	11	11	0	11	22	0.50	0.31
20									
TOTAL	1434	1103	202	113	16	113	331	6.48	6.03
AVERAGE	75.5	58.1	10.6	5.9	0.8	5.9	17.4	0.34	0.32
SEM	3.4	3.0	1.2	0.8	0.2	0.8	1.3	0.04	0.03

p38 Inhibited

#	TOTAL NUMBER OF CELLS							Ratio of PrE/Total ICM	Ratio of Inner/ Outer cells
	Embryo	Double Negative	ICM						
			NANOG +ve	GATA4 +ve	NANOG/ GATA4 double +ve	PrE	Total		
1	52	30	22	0	0	0	22	0.00	0.73
2	51	32	15	4	0	4	19	0.21	0.59
3	67	48	18	1	0	1	19	0.05	0.40
4	58	52	6	0	0	0	6	0.00	0.12
5	55	41	11	1	2	1	14	0.07	0.34
6	77	66	4	7	0	7	11	0.64	0.17
7	84	71	7	2	4	2	13	0.15	0.18
8	61	45	13	3	0	3	16	0.19	0.36
9	65	48	12	5	0	5	17	0.29	0.35
10	25	19	6	0	0	0	6	0.00	0.32
11	53	44	5	3	1	3	9	0.33	0.20
12	63	46	12	3	2	3	17	0.18	0.37
13	97	80	9	8	0	8	17	0.47	0.21
14	60	49	11	0	0	0	11	0.00	0.22
15	81	66	11	4	0	4	15	0.27	0.23
16	74	57	13	4	0	4	17	0.24	0.30
17	67	58	9	0	0	0	9	0.00	0.16
18	81	58	11	12	0	12	23	0.52	0.40
19	74	65	7	1	1	1	9	0.11	0.14
20	106	90	9	7	0	7	16	0.44	0.18
TOTAL	1351	1065	211	65	10	65	286	4.16	5.96
AVERAGE	67.6	53.3	10.6	3.3	0.5	3.3	14.3	0.21	0.30
SEM	4.0	3.8	1.0	0.7	0.2	0.7	1.1	0.04	0.03

Supplementary table S3: Quantification of total and NANOG and/or GATA6 expressing cell numbers for all analysed embryos in KSOM +/- amino acids + DMSO or SB220025 + 1 or 10mM NAC

KSOM

DMSO

#	TOTAL NUMBER OF CELLS						Ratio of NANOG-GATA6 double +ve/Total ICM
			ICM				
	Embryo	Double Negative	NANOG +ve	NANOG-GATA6 double +ve	PrE	Total	
1	75	63	4	6	5	12	0.50
2	65	51	12	9	2	14	0.64
3	70	60	5	4	3	10	0.40
4	65	52	6	11	2	13	0.85
5	70	52	8	4	10	18	0.22
6	97	78	11	8	5	19	0.42
7	89	73	9	4	7	16	0.25
8	82	67	11	6	2	15	0.40
9	92	80	10	3	1	12	0.25
10	82	55	15	8	10	27	0.30
11	87	60	24	23	3	27	0.85
12	88	65	9	7	11	23	0.30
13							
14							
15							
TOTAL	962	756	124	93	61	206	5.38
AVERAGE	80.2	63.0	10.3	7.8	5.1	17.2	0.45
SEM	3.1	2.9	1.5	1.5	1.0	1.7	0.06

p38 Inhibited

#	TOTAL NUMBER OF CELLS						Ratio of NANOG-GATA6 double +ve/Total ICM
			ICM				
	Embryo	Double Negative	NANOG +ve	NANOG-GATA6 double +ve	PrE	Total	
1	70	65	3	5	0	5	1.00
2	37	35	2	2	0	2	1.00
3	75	67	6	5	2	8	0.63
4	38	21	17	17	0	17	1.00
5	38	29	9	9	0	9	1.00
6	90	84	5	2	1	6	0.33
7	45	37	8	8	0	8	1.00
8	27	16	11	11	0	11	1.00
9	40	35	5	5	0	5	1.00
10	37	30	5	7	0	7	1.00
11	52	43	9	9	0	9	1.00
12	45	34	11	11	0	11	1.00
13							
14							
15							
TOTAL	594	496	91	91	3	98	10.96
AVERAGE	49.5	41.3	7.6	7.6	0.3	8.2	0.91
SEM	5.5	5.9	1.2	1.2	0.2	1.1	0.06

KSOM with amino acids

DMSO

#	TOTAL NUMBER OF CELLS						Ratio of NANOG-GATA6 double +ve/Total ICM
			ICM				
	Embryo	Double Negative	NANOG +ve	NANOG-GATA6 double +ve	PrE	Total	
1	84	60	9	0	15	24	0.00
2	86	62	14	11	9	24	0.46
3	89	70	13	4	6	19	0.21
4	72	58	11	8	1	14	0.57
5	58	48	6	2	4	10	0.20
6	73	52	6	2	15	21	0.10
7	85	63	15	7	5	22	0.32
8	90	67	7	2	15	23	0.09
9	85	67	9	6	7	18	0.33
10	117	98	11	4	7	19	0.21
11	84	74	6	2	4	10	0.20
12	88	69	10	3	8	19	0.16
13	107	89	11	2	7	18	0.11
14	98	68	12	3	16	30	0.10
15	94	81	11	9	1	13	0.69
16	90	67	16	6	7	23	0.26
17	80	60	10	7	8	20	0.35
18	79	57	8	3	12	22	0.14
19	93	71	19	14	3	22	0.64
20	66	45	18	4	3	21	0.19
21	101	74	14	2	11	27	0.07
22	72	55	12	3	5	17	0.18
23	83	57	15	7	9	26	0.27
24	71	40	21	14	5	31	0.45
25	92	73	8	5	7	19	0.26
26	78	58	11	8	3	20	0.40
27	80	60	13	0	7	20	0.00
28	77	56	10	2	11	21	0.10
29	60	40	20	11	0	20	0.55
30	78	69	6	0	3	9	0.00
31	95	75	8	6	9	20	0.30
32	106	88	13	6	4	18	0.33
33	80	62	8	2	8	18	0.11
34	111	82	12	0	17	29	0.00
35	110	88	10	1	12	22	0.05
36	76	59	10	6	7	17	0.35
37	96	76	10	3	10	20	0.15
38							
39							

p38 Inhibited

#	TOTAL NUMBER OF CELLS						Ratio of NANOG-GATA6 double +ve/Total ICM
			ICM				
	Embryo	Double Negative	NANOG +ve	NANOG-GATA6 double +ve	PrE	Total	
1	75	65	7	1	3	10	0.10
2	62	43	16	4	3	19	0.21
3	61	47	12	7	2	14	0.50
4	79	66	9	2	4	13	0.15
5	23	18	5	5	0	5	1.00
6	68	54	9	2	4	14	0.14
7	68	59	5	1	4	9	0.11
8	57	43	9	2	5	14	0.14
9	63	50	13	4	0	13	0.31
10	64	59	5	5	0	5	1.00
11	66	55	10	9	1	11	0.82
12	52	44	4	2	3	8	0.25
13	68	61	7	6	0	7	0.86
14	66	50	15	11	1	16	0.69
15	60	49	7	8	2	11	0.73
16	49	37	12	3	0	12	0.25
17	57	50	7	1	0	7	0.14
18	85	60	16	4	8	25	0.16
19	63	54	8	8	0	9	0.89
20	71	60	11	7	0	11	0.64
21	53	34	18	10	0	19	0.53
22	51	48	3	1	0	3	0.33
23	49	39	9	6	1	10	0.60
24	35	27	7	7	1	8	0.88
25	45	28	17	9	0	17	0.53
26	71	59	8	4	1	12	0.33
27	55	49	5	2	1	6	0.33
28	59	29	30	27	0	30	0.90
29	43	24	19	17	0	19	0.89
30	52	44	5	2	2	8	0.25
31	55	34	20	8	0	21	0.38
32	66	56	10	2	0	10	0.20
33	65	50	11	9	4	15	0.60
34	62	49	11	6	2	13	0.46
35	48	36	12	9	0	12	0.75
36	85	69	6	0	10	16	0.00
37	73	56	14	9	1	17	0.53
38	76	54	11	2	11	22	0.09
39							

40							
**TOTAL**	**3184**	**2438**	**423**	**175**	**281**	**746**	8.89
**AVERAGE**	**86.1**	**65.9**	**11.4**	**4.7**	**7.6**	**20.2**	0.24
**SEM**	**2.3**	**2.2**	**0.6**	**0.6**	**0.7**	**0.8**	0.03

40							
**TOTAL**	**2300**	**1809**	**403**	**222**	**74**	**491**	17.68
**AVERAGE**	**60.5**	**47.6**	**10.6**	**5.8**	**1.9**	**12.9**	0.47
**SEM**	**2.1**	**2.0**	**0.9**	**0.8**	**0.4**	**0.9**	0.05

Supplementary table S3 (contd.)

KSOM + 1mM NAC

DMSO

#	TOTAL NUMBER OF CELLS						Ratio of NANOG-GATA6 double +ve/Total ICM
			ICM				
	Embryo	Double Negative	NANOG +ve	NANOG-GATA6 double +ve	PrE	Total	
1	97	75	13	4	8	22	0.18
2	73	51	17	17	2	22	0.77
3	70	43	14	5	10	27	0.19
4	91	68	13	4	10	23	0.17
5	62	50	7	5	2	12	0.42
6	75	47	18	7	9	28	0.25
7	91	73	10	10	7	18	0.56
8	64	52	6	6	6	12	0.50
9	84	71	8	3	4	13	0.23
10	77	58	11	3	8	19	0.16
11	84	70	9	2	5	14	0.14
12							
**TOTAL**	**868**	**658**	**126**	**66**	**71**	**210**	3.57
**AVERAGE**	**78.9**	**59.8**	**11.5**	**6.0**	**6.5**	**19.1**	0.32
**SEM**	**3.5**	**3.5**	**1.2**	**1.3**	**0.9**	**1.8**	0.06

p38 Inhibited

#	TOTAL NUMBER OF CELLS						Ratio of NANOG-GATA6 double +ve/Total ICM
			ICM				
	Embryo	Double Negative	NANOG +ve	NANOG-GATA6 double +ve	PrE	Total	
1	78	67	11	8	0	11	0.73
2	83	81	2	0	0	2	0.00
3	81	70	7	2	4	11	0.18
4	47	46	1	0	0	1	0.00
5	45	38	7	7	0	7	1.00
6	75	67	4	0	4	8	0.00
7	65	61	2	0	2	4	0.00
8	60	51	9	6	0	9	0.67
9	70	63	4	2	3	7	0.29
10	60	51	9	7	0	9	0.78
11							
12							
**TOTAL**	**664**	**595**	**56**	**32**	**13**	**69**	3.64
**AVERAGE**	**66.4**	**59.5**	**5.6**	**3.2**	**1.3**	**6.9**	0.36
**SEM**	**4.3**	**4.1**	**1.1**	**1.1**	**0.6**	**1.1**	0.12

KSOM + 10mM NAC

DMSO

#	TOTAL NUMBER OF CELLS						Ratio of NANOG-GATA6 double +ve/Total ICM
			ICM				
	Embryo	Double Negative	NANOG +ve	NANOG-GATA6 double +ve	PrE	Total	
1	103	83	10	2	8	20	0.10
2	81	60	10	3	11	21	0.14
3	82	61	9	4	11	21	0.19
4	95	78	7	2	10	17	0.12
5	83	72	8	0	3	11	0.00
6	93	75	9	3	9	18	0.17
7	83	66	9	1	8	17	0.06
8	102	86	7	0	9	16	0.00
9	72	48	12	1	12	24	0.04
10							
**TOTAL**	**794**	**629**	**81**	**16**	**81**	**165**	0.82
**AVERAGE**	**88.2**	**69.9**	**9.0**	**1.8**	**9.0**	**18.3**	0.09
**SEM**	**3.5**	**4.1**	**0.5**	**0.5**	**0.9**	**1.2**	0.02

p38 Inhibited

#	TOTAL NUMBER OF CELLS						Ratio of NANOG-GATA6 double +ve/Total ICM
			ICM				
	Embryo	Double Negative	NANOG +ve	NANOG-GATA6 double +ve	PrE	Total	
1	68	55	9	3	4	13	0.23
2	90	74	8	1	8	16	0.06
3	69	61	8	6	0	8	0.75
4	78	59	13	7	6	19	0.37
5	62	52	10	7	0	10	0.70
6	47	39	6	4	2	8	0.50
7	81	67	8	2	6	14	0.14
8	72	64	7	7	1	8	0.88
9	52	45	5	3	2	7	0.43
10	36	25	8	3	3	11	0.27
**TOTAL**	**655**	**541**	**82**	**43**	**32**	**114**	4.33
**AVERAGE**	**65.5**	**54.1**	**8.2**	**4.3**	**3.2**	**11.4**	0.43
**SEM**	**5.2**	**4.6**	**0.7**	**0.7**	**0.9**	**1.3**	0.09

Supplementary table S3 (contd. 2)

KSOM+AA + 1mM NAC

DMSO

#	TOTAL NUMBER OF CELLS						Ratio of NANOG-GATA6 double +ve/Total ICM
			ICM				
	Embryo	Double Negative	NANOG +ve	NANOG-GATA6 double +ve	PrE	Total	
1	97	75	12	3	9	22	0.14
2	87	77	7	9	1	10	0.90
3	49	33	10	13	3	16	0.81
4	45	30	11	13	1	15	0.87
5	68	56	9	9	2	12	0.75
6	78	50	17	6	11	28	0.21
7	78	62	10	3	5	16	0.19
8	56	53	3	1	0	3	0.33
9	68	45	16	12	5	23	0.52
10	91	73	6	0	12	18	0.00
11	78	57	13	6	8	21	0.29
12	89	68	5	4	12	21	0.19
13	93	77	9	2	7	16	0.13
14	88	63	6	3	18	25	0.12
15	73	47	13	11	9	26	0.42
16	67	46	19	12	2	21	0.57
17	79	67	9	3	3	12	0.25
18	100	76	13	4	11	24	0.17
19	93	75	9	6	8	18	0.33
20	90	66	17	10	7	24	0.42
21							
22							
23							
24							
25							
**TOTAL**	**1567**	**1196**	**214**	**130**	**134**	**371**	7.60
**AVERAGE**	**78.4**	**59.8**	**10.7**	**6.5**	**6.7**	**18.6**	0.38
**SEM**	**3.5**	**3.2**	**1.0**	**1.0**	**1.1**	**1.4**	0.06

p38 Inhibited

#	TOTAL NUMBER OF CELLS						Ratio of NANOG-GATA6 double +ve/Total ICM
			ICM				
	Embryo	Double Negative	NANOG +ve	NANOG-GATA6 double +ve	PrE	Total	
1	79	68	5	0	6	11	0.00
2	57	43	12	2	2	14	0.14
3	63	42	15	3	5	21	0.14
4	66	57	9	4	0	9	0.44
5	60	45	12	5	3	15	0.33
6	81	73	2	0	6	8	0.00
7	95	79	9	1	6	16	0.06
8	59	46	13	5	0	13	0.38
9	13	8	5	0	0	5	0.00
10	76	68	3	0	5	8	0.00
11	28	11	17	14	0	17	0.82
12	96	84	8	3	4	12	0.25
13	76	61	9	2	4	15	0.13
14	71	55	16	8	0	16	0.50
15	45	37	8	6	0	8	0.75
16	58	44	14	12	0	14	0.86
17	85	72	13	9	0	13	0.69
18	60	47	11	9	2	13	0.69
19	80	71	6	3	3	9	0.33
20	63	55	7	5	1	8	0.63
21	62	49	5	1	8	13	0.08
22	22	19	3	2	0	3	0.67
23							
24							
25							
**TOTAL**	**1395**	**1134**	**202**	**94**	**55**	**261**	7.91
**AVERAGE**	**63.4**	**51.5**	**9.2**	**4.3**	**2.5**	**11.9**	0.36
**SEM**	**4.6**	**4.4**	**0.9**	**0.8**	**0.6**	**0.9**	0.06

KSOM+AA + 10mM NAC

DMSO

#	TOTAL NUMBER OF CELLS						Ratio of NANOG-GATA6 double +ve/Total ICM
			ICM				
	Embryo	Double Negative	NANOG +ve	NANOG-GATA6 double +ve	PrE	Total	
1	84	61	17	4	6	23	0.17
2	64	54	9	4	1	10	0.40
3	66	41	21	9	3	25	0.36
4	66	40	21	8	1	26	0.31
5	95	67	16	1	12	28	0.04
6	58	38	16	11	3	20	0.55
7	92	76	6	0	10	16	0.00
8	45	35	5	1	4	10	0.10
9	70	55	7	1	7	15	0.07
10	91	75	9	0	7	16	0.00
11	93	78	8	4	6	15	0.27
12	84	64	8	2	11	20	0.10
13	85	65	10	4	8	20	0.20
14	69	59	10	10	0	10	1.00
15	71	56	7	3	6	15	0.20
16	94	72	11	1	11	22	0.05
17							
18							
19							
20							
**TOTAL**	**1227**	**936**	**181**	**63**	**96**	**291**	3.81
**AVERAGE**	**76.7**	**58.5**	**11.3**	**3.9**	**6.0**	**18.2**	0.24
**SEM**	**3.8**	**3.5**	**1.3**	**0.9**	**0.9**	**1.4**	0.06

p38 Inhibited

#	TOTAL NUMBER OF CELLS						Ratio of NANOG-GATA6 double +ve/Total ICM
			ICM				
	Embryo	Double Negative	NANOG +ve	NANOG-GATA6 double +ve	PrE	Total	
1	25	19	6	2	0	6	0.33
2	53	44	5	1	3	9	0.11
3	63	46	12	3	3	17	0.18
4	97	80	9	0	8	17	0.00
5	60	49	11	7	0	11	0.64
6	81	66	11	3	4	15	0.20
7	74	57	13	2	4	17	0.12
8	67	58	9	5	0	9	0.56
9	81	58	11	0	12	23	0.00
10	74	65	7	4	1	9	0.44
11	106	90	9	0	7	16	0.00
12							
13							
14							
15							
16							
17							
18							
19							
20							
**TOTAL**	**781**	**632**	**103**	**27**	**42**	**149**	2.57
**AVERAGE**	**71.0**	**57.5**	**9.4**	**2.5**	**3.8**	**13.5**	0.23
**SEM**	**6.6**	**5.7**	**0.8**	**0.7**	**1.2**	**1.5**	0.07

Supplementary table S4 (antibodies used): Antibodies used for immunofluorescence staining and western blotting

Sl. No.	Antibody	Type	Target	Host / isotype	Manufacturer	Catalogue number	Dilution used
**Immunofluorescence imaging**							
1	Nanog (eBioMLC-51)	Primary; monoclonal	Mouse	Rat / IgG2a	Thermo Fisher Scientific Inc. (eBioscience™)	14-5761-80	1:200 in PBST (3% BSA)
2	GATA-4 (H-112)	Primary; polyclonal	Mouse, rat and human	Rabbit / IgG	Santa Cruz Biotechnology, Inc.	sc-9053	1:200 in PBST (3% BSA)
3	GATA-6	Primary; polyclonal	Human and mouse	Goat / IgG	R&D Systems™	AF1700	1:200 in PBST (3% BSA)
4	Donkey anti-Rat IgG (H+L) Highly Cross-Adsorbed Secondary Antibody, Alexa Fluor 488	Secondary; polyclonal	Rat	Donkey / IgG	Thermo Fisher Scientific Inc.	A-21208	1:500 in PBST (3% BSA)
5	Donkey anti-Rabbit IgG (H+L) Highly Cross-Adsorbed Secondary Antibody, Alexa Fluor 555	Secondary; polyclonal	Rabbit	Donkey / IgG	Thermo Fisher Scientific Inc.	A-31572	1:500 in PBST (3% BSA)
6	Donkey Anti-Goat IgG H&L (Alexa Fluor® 647)	Secondary; polyclonal	Goat	Donkey / IgG	Abcam plc.	ab150131	1:500 in PBST (3% BSA)
7	Donkey Anti-Rabbit IgG H&L (Alexa Fluor® 647)	Secondary; polyclonal	Rabbit	Donkey / IgG	Abcam plc.	ab150075	1:500 in PBST (3% BSA)
8	Goat Anti-Rat IgG H&L (Cy3 ®) preadsorbed	Secondary; polyclonal	Rat	Goat / IgG	Abcam plc.	ab98416	1:500 in PBST (3% BSA)
**Immunoblotting**							
9	Phospho-p38 MAPK (Thr180/Tyr182) (28B10)	Primary; monoclonal	Mouse, rat and human	Mouse / IgG1	Cell Signaling Technology, Inc.	9216S	1:500 in 1% milk
10	GAPDH	Primary; polyclonal	Mouse, rat and human	Rabbit	Merck KGaA (Sigma-Aldrich, Inc.)	G9545	1:20,000 in 1% milk
11	Peroxidase AffiniPure Goat Anti-Mouse IgG (H+L)	Secondary; polyclonal	Mouse	Goat / IgG	Jackson ImmunoResearch Europe Ltd.	115-035-003	1:10,000 in 1% milk
12	Peroxidase AffiniPure Donkey Anti-Rabbit IgG (H+L)	Secondary; polyclonal	Rabbit	Donkey / IgG	Jackson ImmunoResearch Europe Ltd.	711-035-152	1:10,000 in 1% milk

Supplementary table S5 (ANOVA results for figures 4 and 5e & e'):
Welch's ANOVA test results (corresponding to figures 4, 5e and e')

Welch's ANOVA test	KSOM		KSOM+AA	
	DMSO	SB220025 (p38i)	DMSO	SB220025 (p38i)
**Total cell number**	0.5568 (ns)	0.0005 (***)	0.2422 (ns)	0.1291 (ns)
**Outer cell number**	0.6594 (ns)	0.0019 (**)	0.2643 (ns)	0.2595 (ns)
**Inner cell number**	0.6266 (ns)	0.0229 (*)	0.7817 (ns)	0.1476 (ns)
**Epiblast cell number**	0.0959 (ns)	0.1745 (ns)	0.9893 (ns)	0.5693 (ns)
**PrE cell number**	0.9329 (ns)	0.0022 (**)	0.5677 (ns)	0.2169 (ns)
**PrE to ICM ratio**	0.6893 (ns)	0.0015 (**)	0.6342 (ns)	0.2126 (ns)
**Uncommitted to ICM ratio**	<0.0001 (****)	0.0002 (***)	0.1308 (ns)	0.0348 (*)

Supplementary table S6 (oligonucleotide primers used for Q-RT PCR; Fig 3h):
Oligonucleotide primers used for Q-RT PCR (corresponding to figure 3h)

Sequences of oligonucleotide primer pairs used for Q-RT PCR			
**Sl. No.**	**Gene**	**Forward primer (5'-3')**	**Reverse primer (5'-3')**
1	H2afz	GCGCAGCCATCCTGGAGTA	CCGATCAGCGATTTGTGGA
2	Cat	TCCTCGTTCAGGATGTGGTT	TGCGTGTACCACTCTCTCAG
3	Sod1	CGGTGAACCAGTTGTGTTGT	GGTCTCCAACATGCCTCTCT
4	Sod2	CAAGCACAGCCTCCCAGA	ATCTGCGCGTTAATGTGTGG

Supplementary Fig. S1 (supplementary to Fig 3 b (fixed))

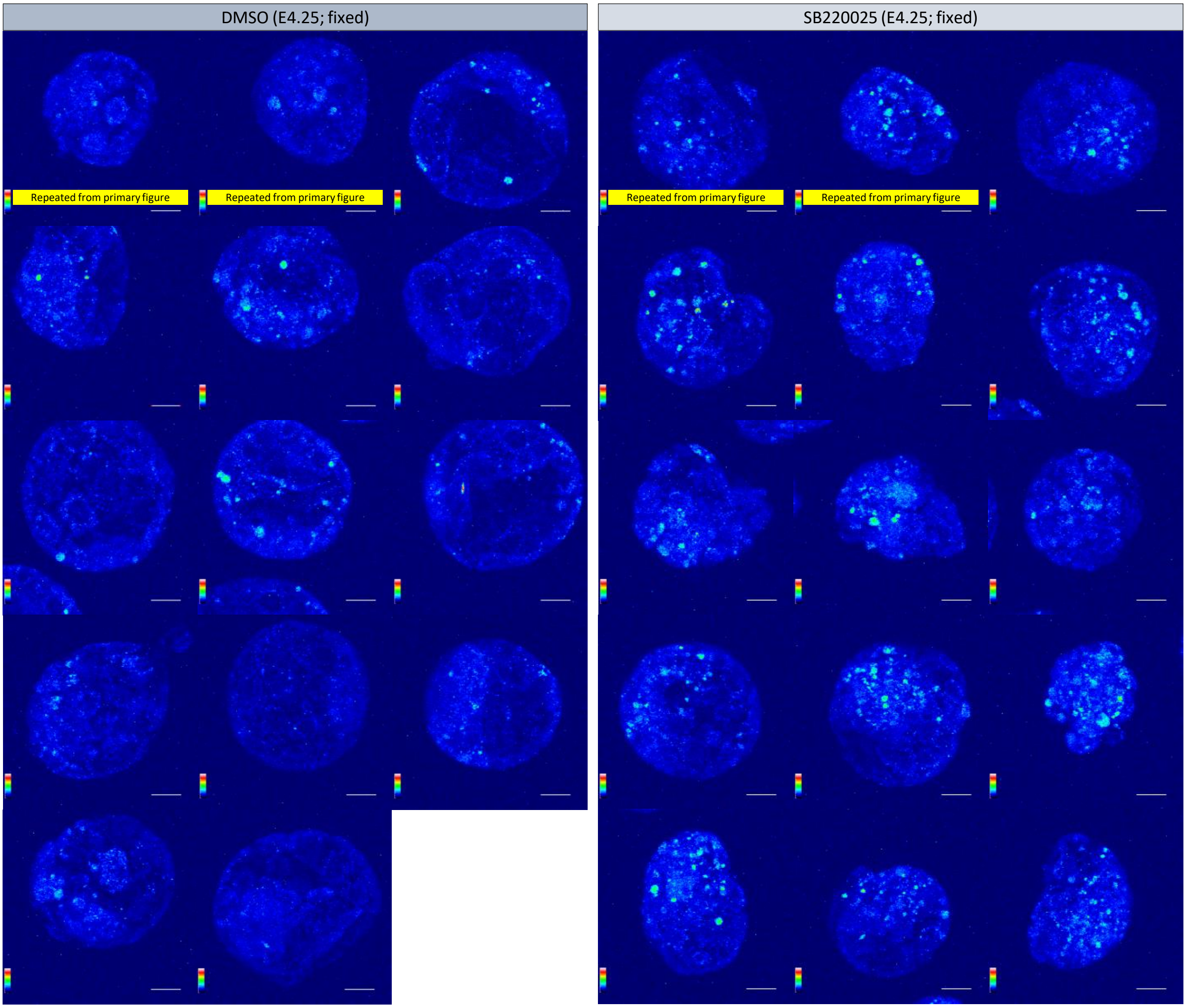

(Supplementary table to Fig 3 c)

Count of ROX-positive foci (maxima) for E4.25

	KSOM	
	DMSO	SB220025
	204	442
	291	422
	314	466
	456	462
	543	617
	423	541
	393	589
	591	623
	385	478
	393	520
	144	668
	461	608
	389	640
	237	346
		567
Average	373.14	532.60
